# Exploring the use and impact of adjuvant Trastuzumab for HER2-positive breast cancer patients in a large UK cancer network Do the results of international clinical trials translate into a similar benefit for patients in South East Wales?

**DOI:** 10.1038/bjc.2011.506

**Published:** 2011-11-22

**Authors:** R M Webster, J Abraham, N Palaniappan, A Caley, B Jasani, P Barrett-Lee

**Affiliations:** 1 Department of Clinical Oncology, Velindre Cancer Centre, Whitchurch, Cardiff CF14 2TL, UK; 2 Department of Pathology, University Hospital of Wales, Cardiff, UK

**Keywords:** trastuzumab, adjuvant, HER2+

## Abstract

**Background:**

Trastuzumab was approved in the United Kingdom for adjuvant treatment of human epidermal growth factor receptor 2 (HER2)+ breast cancer in 2006 at significant economic cost and with limited evidence in smaller T1N0 tumours. The South East Wales Cancer Network covers a population of 1 420 000 and maintains a database of treatments used. We examined this database to ensure the outcome of Trastuzumab use is as expected, especially in patients with T1N0 cancers.

**Ethods:**

M Case notes of patients with HER2+ disease eligible for adjuvant Trastuzumab over 2005–2008 were reviewed. Disease-free survival (DFS) and overall survival (OS) were calculated with the Kaplan–Meier method using SPSS (version 16.0.01 for Windows, SPSS, Chicago, IL, USA).

**Results:**

A total of 239 of 338 (70.7%) eligible HER2+ patients received treatment. At 3 years, the DFS of the treated group was 90.3% *vs* 73.3% and the OS was 98.5% *vs* 87.6%. In all, 47 of 92 stage I patients received Trastuzumab. Despite a trend towards worse prognostic factors in the treated group the DFS was 100% *vs* 84.1% and the OS was 100% *vs* 93.3%.

**Conclusion:**

Our results are comparable to those from landmark Trastuzumab trials. As evidence continues to emerge that smaller HER2+ cancers may behave aggressively our analysis of stage I tumours adds further support to the use of Trastuzumab in these patients.

Breast cancer is the most common cancer in the United Kingdom with almost 48 000 new cases presenting in 2008 (CRUK, 2011). Between 15% and 20% of breast cancers show overexpression or gene amplification of the human epidermal growth factor receptor 2 (HER2), a trans-membrane receptor protein at the head of an intracellular tyrosine kinase signalling cascade. Activation of this pathway (‘HER2+’) results in increased proliferation, increased anti-apoptotic protein expression and angiogenesis, and is associated with worse prognosis (Slamon *et al*, 1987). Geographically, the rate of HER2+ varies and has recently been reported as 15.1% in our region (Webster *et al*, 2010). Trastuzumab, an anti-HER2 monoclonal antibody, was approved as an adjuvant treatment for HER2+ breast cancer by the National Institute of Clinical Excellence (NICE) in August 2006. As a result, all HER2+ breast cancer patients with a primary tumour of over 10 mm are eligible for this treatment in the adjuvant setting and its use has become the standard of care, although at a significant financial cost (NICE, 2006).

Trastuzumab was approved following the publication of several international phase III randomised trials showing a statistically significantly improvement in disease-free survival (DFS) when used for 1 year following adjuvant chemotherapy (Viani *et al*, 2007). The HERA study involved many UK cancer centres and the results are most applicable to UK patients (Piccart-Gebhart *et al*, 2005). In March 2011, the updated 4-year outcome data from this trial were published showing a statistically significant improvement in DFS at 4 years from 72.2% to 78.6%, but without an overall survival (OS) benefit to date (Gianni *et al*, 2011). Meta-analysis of several similar landmark trials (HERA, Piccart-Gebhart *et al*, 2005; BCIRG 006, Slamon *et al*, 2006; NSABP B-31, Romond *et al*, 2005; Intergroup N9831, Romond *et al*, 2005) reveals a consistent improvement in DFS and OS (Viani *et al*, 2007).

One area of current debate is the role of adjuvant Trastuzumab in patients with node-negative HER2+ cancers with a diameter of 10 mm or less (T1aN0 or T1bN0—TNM 7th edition; Sobin *et al*, 2009) who are traditionally thought to be at a low risk of recurrence (Banerjee and Smith, 2010). This is increasingly relevant with the development of more sensitive mammographic screening tools and the expanding use of MRI scanning, resulting in a higher proportion of patients diagnosed at an earlier stage and before nodal disease has developed (Schmidt *et al*, 1998). The use of adjuvant treatment with a combination of radiotherapy, chemotherapy, hormone therapy and biological agents is determined by an assessment of the patient, assessment of the pathological features of the tumour, and an estimate of the absolute reduction in the risk of recurrence that these agents can bring. Unfortunately, many of the adjuvant Trastuzumab trials excluded HER2+ T1aN0 or T1bN0 cancers because of the perceived low risk of recurrence and there remains a lack of robust evidence which can be used to estimate the balance of benefits and risks of biological therapy in these patients (Banerjee and Smith, 2010).

Retrospective studies suggest that HER2+ patients with T1aN0 or T1bN0 tumours have a significantly worse survival than HER2− patients with similar tumour characteristics (Banerjee and Smith, 2010). One large retrospective case review of 965 patients with T1aN0 orT1bN0 breast cancers, (10% of which were HER2+) showed that the HER2+ patients had a significantly worse recurrence-free survival compared with HER2− cases (77.1% *vs* 93.7% at 5 years *P*<0.0001; Gonzalez-Angulo *et al*, 2009). Several other studies have reported a similar increased risk of recurrence (Curigliano *et al*, 2009; Tovey *et al*, 2009; Banerjee and Smith, 2010).

Extrapolation of the benefits demonstrated in the adjuvant Trastuzumab trials and evidence from small case series of HER2+ patients with T1aN0 and T1bN0 tumours suggest there may be a similar relative benefit from biological therapies regardless of stage and nodal status, indicating that if the worse survival of these tumours is correct there would be a significant absolute benefit from treating these patients with adjuvant Trastuzumab (Banerjee and Smith, 2010; Rodrigues *et al*, 2010). Balancing this uncertain absolute benefit and potential harm of treatment in individual patients remains a significant problem in clinical practice (Kelly *et al*, 2011).

The South East Wales Cancer Network (SEWCN), based at Velindre Cancer Centre, Cardiff, serves a population of 1.42 million patients in South East Wales and maintains an electronic database of patients who have been treated with Trastuzumab. We have analysed the information from this database for two reasons: first, to ensure that our use of Trastuzumab and our outcome data are comparable to published results and NICE guidance; and second, to use this large data set in an attempt to demonstrate a benefit, if any, of adjuvant Trastuzumab in our HER2+ patients with node-negative T1a or T1b tumours.

## Materials and methods

All patients in the SEWCN diagnosed with breast cancer undergo centralised HER2 status testing as part of routine histopathological assessment at the University Hospital of Wales, Cardiff, a UK NEQAS member and regional reference laboratory. Over the period of this retrospective study (2005–2008), the University Hospital of Wales histopathology department defined HER2+ as grade 3 on immunohistochemical (IHC) criteria, or as grade 2 on IHC but with HER2-positive status on FISH analysis (i.e., HER2/CEP17 ratio >2.0). Using the histopathology department records, we identified all HER2+ breast cancer specimens tested over a 4-year period (from 1 January 2005 to 31 December 2008). The start date was chosen to cover the introduction of Trastuzumab into clinical use, including the period before formal NICE approval when Trastuzumab was used in the SEWCN following individual patient-based applications for local funding. In view of the prolonged duration of adjuvant treatments involved a cutoff date of 31 December 2008 was chosen to allow the accrual of adequate clinical follow-up.

**Figure 1:**
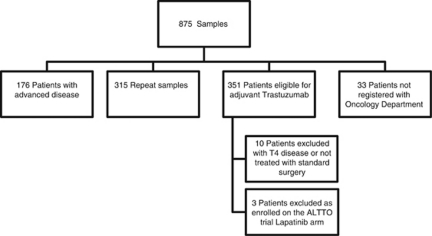
875 HER2+ samples identified from histopathology department records and the corresponding patient information.

A total of 875 HER2+ specimens were identified (Figure 1). For several reasons 315 samples were duplicates, including dual registration for different local hospitals in the SEWCN, testing of different tumours from the same patient, or testing of recurrences from the same patient, which occurred during follow-up after adjuvant treatment. The SEWCN electronic case records of all patients referred for treatment in the network are maintained at Velindre Cancer Centre, Cardiff. The notes were reviewed (RW, NP, AC). In all, 176 patients with HER2+ samples had been referred for testing for treatment with palliative intent. Thirty-three patients had been discussed at the regional MDT and were not referred for further adjuvant treatment (the main reasons being because of age or clinically significant co-morbidity). In total, 351 patients were referred for consideration of adjuvant Trastuzumab. We excluded three patients who received concurrent anti-HER2 therapy as part of the ALTTO trial (study no. BIG 2-06/N06D/EGF106708). We excluded a further 10 patients with T4 disease or who declined standard surgical treatments, leaving 338 eligible patients for further analysis (Figure 1).

Data from the electronic case notes were recorded including patient demographics, dates and type of surgery, tumour characteristics, and all adjuvant treatments used. The use of Trastuzumab was recorded, as were the decisions made as to why treatment was given or not used. All patients who received Trastuzumab were identified, including the number of treatments received and reasons for ending treatment. The date of tumour recurrence or death was taken from the first date documented in the electronic case notes. The DFS was calculated from the date of initial surgery to the date of loco-regional recurrence, distant recurrence, or death from any cause. Overall survival was defined as death from any cause taken from the date of surgery. The date of surgery was chosen as the initial time point to allow comparison of Trastuzumab-treated patients with untreated patients in the absence of a randomisation date, and is roughly comparable to the date of randomisation used for analysis in the BCIRG 006 (Slamon *et al*, 2006), NSABP B-31 (Romond *et al*, 2005), and Intergroup N9831 (Romond *et al*, 2005) trials. Randomisation in the HERA study occurred following four cycles of adjuvant chemotherapy and the results may be less applicable to our analysis (Piccart-Gebhart *et al*, 2005). Patients without evidence of recurrence were censored at the date of the last electronic record compatible with survival. SPSS version 16.0.01 for Windows, SPSS (Chicago, IL, USA) was used to calculate survival times and generate survival curves using the Kaplan–Meier method and comparisons were made using the log-rank test.

## Results

Using the methods outlined above, we identified a total of 338 patients during the 4-year period 1 January 2005 to 31 December, 20 as having undergone surgery for stage I–III HER2+ breast cancer and referred for consideration of adjuvant therapy with Trastuzumab (Figure 1). The median age at diagnosis was 56 years (range of 27–85 years). The median duration of follow-up from the date of surgery was 37.3 months.

**Figure 2:**
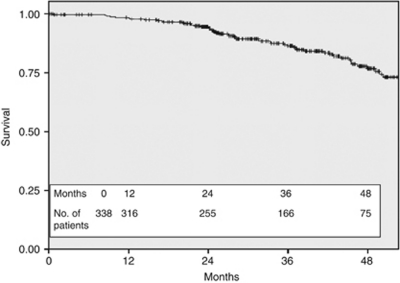
Disease-free survival for all 338 HER2+ eligible patients.

**Figure 3:**
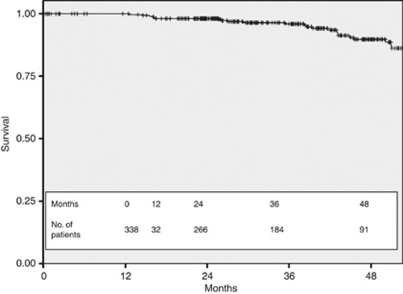
Overall survival for all HER2+ eligible patients.

The histological features of the tumours from the 338 patients are shown in Table 1. The majority of patients had early stage disease with 92 (27.2%) having stage I tumours, 148 (43.8%) stage II and 96 (28.4%) stage III cancers (TNM seventh edition; Sobin *et al*, 2009). In 2 (0.6%) patients, the full pathological tumour stage was unobtainable as they refused surgical axillary nodal staging and were treated with axillary radiotherapy, although on clinical and radiological assessment they were documented as node negative. On histological assessment, 303 (89.6%) cancers were invasive ductal carcinomas, 18 were invasive lobular cancers, and the rest were of mixed pathology. The histological grade was grade 3 in 226 (66.9%) patients, 104 (30.8%) were grade 2, and only 7 (2.0%) were grade 1 cancers (the grade of one sample was not obtainable as the histology showed non-gradable micro-invasive disease). The hormone receptor expression, defined as sufficiently positive to warrant the recommendation of treatment with adjuvant hormone therapy, was positive in 207 (61%) and negative in 131 patients (39%). Of the total cohort of 338 eligible patients, the DFS at 36 months from the date of surgery was 86.4% (Figure 2) and the OS was 95.9% at 36 months (Figure 3).

In total, 239 of the 338 patients (70.7%) were treated with adjuvant Trastuzumab. Every patient treated with Trastuzumab had received prior adjuvant chemotherapy. A taxane containing chemotherapy regime was used in 58 patients, of which 4 had taxane chemotherapy and Trastuzumab concurrently. The remaining 54 had treatment sequentially. Hormone therapy and appropriate local therapy with whole breast radiotherapy were given as indicated. In general, the majority of patients with the higher stage cancers received adjuvant treatment with chemotherapy and Trastuzumab, while progressively fewer lower stage patients were treated (Table 2). Of 96 patients with stage III cancers, 78 (81.3%) were treated with Trastuzumab, decreasing to 112 of 148 (75.7%) stage II patients and 47 of 92 (51.1%) stage I patients. The two patients who refused surgical treatment to the axilla (and did not have a pathological stage) both received adjuvant Trastuzumab. Only 28 of the 99 (28.3%) patients who did not receive Trastuzumab were treated with chemotherapy (6 stage I, 13 stage II, and 9 stage III). The reason no Trastuzumab was given because cardiac co-morbidity in 13 (46.4%), 7 (25.0%) declined Trastuzumab, 3 (10.7%) because of lack of funding in the period before NICE approval and 2 (7.4%) because recurrence developed before Trastuzumab (one patient with lung metastasis and one with contralateral breast cancer stage T1N1M0). The other three patients received chemotherapy but Trastuzumab was not recommended because of a perceived low recurrence risk by the treating clinician.

A full-prescribed course of treatment with 18 cycles of Trastuzumab, administered once every 21 days, was completed by 194 of 239 patients (81.2%). A fall in ejection fraction resulted in termination of treatment in 33 patients (13.8%). In 2005, 7 of 41 (17.1%) terminated treatment because of a fall in ejection fraction increasing to 18 of 81 (22.2%) in 2006. Subsequently, the rate of Trastuzumab termination for cardiac reasons decreased in the final 2 years of the study with 5 of 59 (8.5%) patients stopping early in 2007 and 3 of 58 (5.2%) in 2008.

An asymptomatic fall in ejection fraction that failed to recover adequately with cessation of treatment and medical treatment occurred in 21 patients. Symptomatic falls in cardiac function occurred in 12 patients, 10 of whom recovered with medical treatment and cessation of Trastuzumab, and in all of these patients Trastuzumab was not recommenced. Only 1 of the 12 symptomatic patients developed clinical signs of significant cardiac failure and continued to be symptomatic 2 years later. One patient with a symptomatic fall in ejection fraction recovered adequately enough to receive further treatment with Trastuzumab in the palliative setting after developing recurrent disease 19 months later. After a further 18 months of Trastuzumab, the patient developed cardiac failure requiring termination of Trastuzumab therapy and eventually died as a consequence of metastatic disease.

Disease recurrence during treatment resulted in four (1.7%) patients terminating the prescribed course. Other reasons for stopping include allergic reactions (two), recurrent line infection (two), pregnancy (one), depression (one), and moving out of area resulting in being lost to follow-up (two).

**Figure 4:**
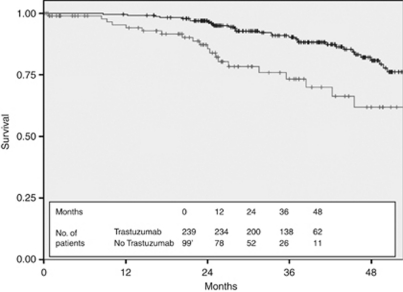
Disease-free survival for all HER2+ patients stratified by Trastuzumab treatment (black—treated with Trastuzumab and grey—not treated with Trastuzumab).

**Figure 5:**
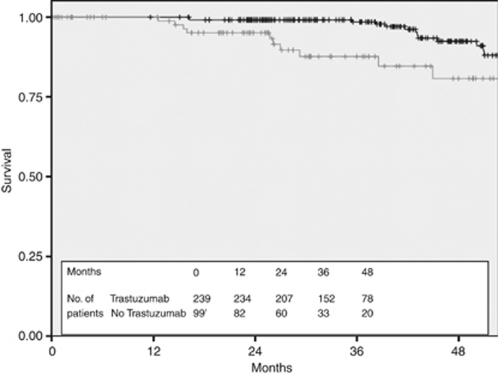
Overall survival for all HER2+ eligible patients stratified by Trastuzumab use (black—treated with Trastuzumab and grey—not treated with Trastuzumab).

**Figure 6:**
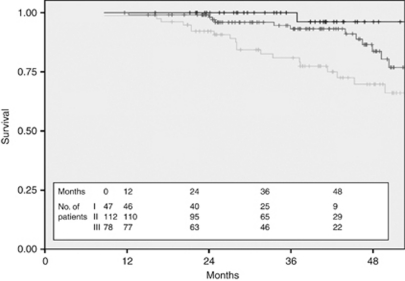
Disease-free survival by stage for HER2+ patients treated with Trastuzumab (black—stage I, dark grey—stage II, and light grey—stage III).

**Figure 7:**
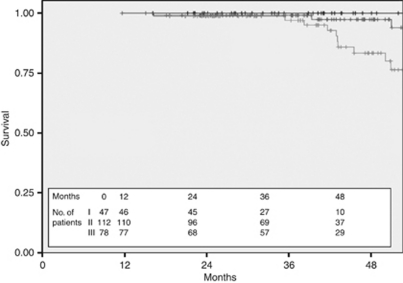
Overall survival by stage for HER2+ patients treated with Trastuzumab (black—stage I, dark grey—stage II, and light grey—stage III).

The 3-year DFS of the 239 patients treated with adjuvant Trastuzumab was 90.3% compared with 73.3% in the 99 patients in the untreated cohort (Figure 4) and the 3-year OS was 98.5% compared with 87.6% (Figure 5). Stratification of the treated cohort of 237 pathologically staged patients by stage reveals a 3-year DFS of 100% in the 47 treated stage I patients, compared with 93.2% in the 112 treated stage II patients and 77.4% in the 78 stage III patients (Figure 6). The corresponding 3-year OS is 100% in stage I patients, 99.1% in stage II patients, and 97% in stage III patients (Figure 7).

**Figure 8:**
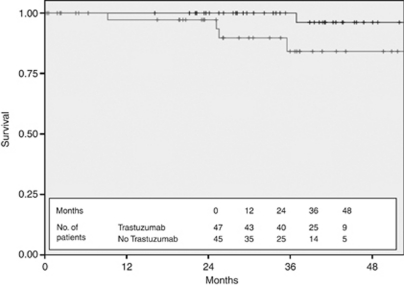
Disease-free survival for stage I patients stratified by Trastuzumab use (black—treated with Trastuzumab and grey—not treated with trastuzumab).

**Figure 9:**
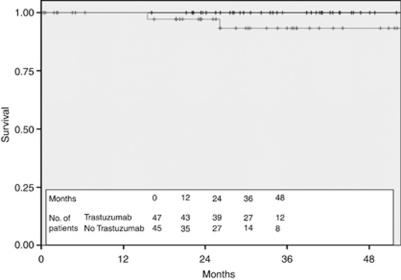
Overall survival for stage I patients stratified by Trastuzumab use (black—treated with Trastuzumab and grey—not treated with Trastuzumab).

There were 92 patients with stage I disease. Adjuvant chemotherapy and Trastuzumab were used in 47 of 92 patients (51%). In the untreated group of 45 patients, 6 received adjuvant chemotherapy but did not have Trastuzumab despite its recommended use by the treating clinician (2 because this was not funded, 3 because of cardiac risk factors, and 1 because the patient declined). The characteristics of the treated and untreated groups are shown in Table 3 and were generally similar, with a trend to better prognostic features in the untreated cohort. Despite the trend towards more favourable disease characteristics in the untreated group, the DFS was significantly worse in the untreated stage I cohort, with a 3-year DFS of 100% in the treated group compared with 84.1% in the untreated cohort (Figure 8) (*P*=0.059 using the log-rank test). None of the 47 treated patients died during follow-up compared with 2 of the 45 untreated patients (1 died as a result of distant metastatic breast cancer 15 months after initial diagnosis and 1 from small cell lung cancer 26 months after initial breast cancer diagnosis), resulting in an OS at 3 years of 100% in the treated arm and 93.3% in the untreated arm (Figure 9) (*P*=0.09 using long-rank test).

This stage I cohort included 67 patients with T1cN0 disease, of which 27 were not treated with adjuvant Trastuzumab. Chemotherapy was declined by three patients with T1cN0 disease. Systemic chemotherapy but not Trastuzumab was given to 6 (2 because funding for Trastuzumab was not available, 3 because of significant cardiac co-morbidities, and 1 because the patient declined). In the remaining 18 patients with T1cN0 disease not treated with chemotherapy and Trastuzumab it was felt by the clinician, after assessing the patients age and co-morbidities, that the risk of treatment outweighed the benefits. The majority of these patients (66%) were hormone receptor positive.

In all, 25 patients (7.4%) of the whole 338 HER2+ patient cohort had node-negative disease with a primary tumour size of 10 mm or less (T1aN0 or T1bN0). Of these patients, seven (28.0%) were treated with adjuvant chemotherapy and Trastuzumab and to date there have been no recurrences in this group (all patients had grade 3 invasive ductal cancers, 6 out of 7 were ER negative). In the 18 untreated patients, 2 have had recurrence, one with loco-regional recurrence 25 months after surgery and one with lung and liver metastasis also at 25 months after diagnosis. However, both of these patients remain alive 12 months following disease recurrence.

## Discussion

The limitations of any retrospective review of practice are considerable and, without prospective randomisation and control for the use of adjuvant chemotherapy in the untreated cohort, the true effects of Trastuzumab cannot be accurately ascertained. However, despite these limitations, in the absence of randomised trial evidence to guide the treatment of small HER2+ cancers there may be some, albeit less robust, evidence to be gained from a review of practice like this. Our data set represents the typical workload over 4 years of HER2+ breast cancer patients in a large cancer network and should be representative of most UK cancer centres. The SEWCN system of electronic case records, electronic chemotherapy treatment records, and accurate recording of event information allows the accurate monitoring of the treatment these patients receive and allows the assessment of their response to new and expensive therapies. The size of our cohort is also sufficient for us to make some inferences about the treatment of small HER2+ early breast cancers; a group for whom randomised trial information is unlikely to ever be available (Banerjee and Smith, 2010).

When assessing the baseline characteristics our data show patterns to be expected of this higher risk group of breast cancer patients. The higher proportion of larger, hormone receptor negative, and higher grade cancers than unselected breast cancer patients is in keeping with the characteristics of more aggressive HER2+ disease. The SEWCN use of Trastuzumab in 70% of eligible patients is consistent with other published UK results, including a recent review of the use of Trastuzumab in the North Trent Cancer Network in which 129 out of 158 HER2+ patients (69.7%) received this drug (Coulson *et al*, 2010). The majority (82.4%) of higher risk patients in our cohort receive Trastuzumab, decreasing to 50% of patients with stage I tumours.

Once commenced on treatment, the majority (81.2%) complete the prescribed course and if treatment is stopped early it is usually because of a fall in left ventricular ejection fraction. Interestingly, the proportion of patients stopping treatment early for cardiac toxicity fell from 22.2% in 2006 to 5.2% in 2008, which may reflect increasing clinician confidence in managing the cardiac effects of Trastuzumab. Similar figures are reproduced in other retrospective series (Murray *et al*, 2010).

The DFS improvement in our treated cohort compared with untreated cohort (DFS at 36 months of 90.3% *vs* 73.3%) is comparable to the combined analysis of Trastuzumab-treated and untreated cohorts in the NSABP B-31 and Intergroup N9831 trials (DFS at 36 months of 87.1% *vs* 75.4%) (Romond *et al*, 2005). Our OS figures (3-year OS 98.5% *vs* 87.6%) are also similar to the NSABP B-31 and Intergroup N9831 combined results (3-year OS 94.3% *vs* 91.7% Romond *et al*, 2005). The results from the HERA trial are also similar, but may be less applicable because the timing of trial randomisation is significantly later than the date of surgery we have used in our analysis (Piccart-Gebhart *et al*, 2005). In general, our results are reassuring and suggest that the effectiveness of this treatment in the ‘real-life’ clinical setting appears to be as expected, albeit within the limitations of this type of retrospective analysis.

The analysis of stage I tumours is interesting and adds further weight to mounting evidence that HER2+ stage I, and more specifically T1aN0 and T1bN0 tumours, which represents 7% of the sample, have a worse outlook than expected. This is demonstrated by a 3-year DFS of 84.1% in the untreated stage I cohort compared with 100% in the chemotherapy and Trastuzumab-treated group, even with a trend towards better prognostic features in the untreated group. Our DFS result for treated stage I patients appears similar to the benefit obtained by a similar cohort published by Rodrigues *et al* (2010) in a retrospective review of Trastuzumab use in T1a,bN0 HER2+ breast cancer. Unfortunately, although the lack of recurrences or deaths in the treated group compared with the untreated cohort is an interesting observation, our study population is too small to make any significant inferences about the value of Trastuzumab treatment in the specific subset of patients with stage I cancers with a primary size of 10 mm or less.

## Conclusions

The approval of Trastuzumab by NICE in 2006 as an adjuvant treatment for HER2+ breast cancer has resulted in the widespread use of this agent, based on the robust evidence from several international randomised trials. It takes several years, even in large cancer network like the SEWCN, for a sufficient number of patients to be treated and for sufficient follow-up to have accrued before it is possible to assess the true impact of new treatments. It becomes especially important to review the impact of new agents in this era of constrained resources and increasingly expensive treatments, and ensure that data from highly motivated, selected, and well-monitored clinical trial patients translate to the ‘real-life’ clinical setting.

The lack of robust information available to guide clinical decisions in the subgroup of patients with T1aN0 or T1bN0 HER2+ tumours remains a significant clinical issue. The low rate of HER2+ in these cancers also limits the power of retrospective reviews such as this. One potential way of improving the information available would be to pool outcome data on these cancers from different centres, or nationally, to improve the statistical power and validity of this type of retrospective analysis. In the future, this issue may also be addressed by the enrolment of patients with these small cancers into clinical trials designed to confirm the prognostic impact of HER2 status and the benefits of adjuvant Trastuzumab within the different hormone receptor groups, tumour grades, and proliferative indices (Joerger *et al*, 2011).

Despite the limitations of this type of study, our data demonstrate reproducible survival figures compared with relevant trial data, supporting the ongoing use of adjuvant Trastuzumab in the SEWCN HER2+ population. Even with the relatively small numbers of patients with stage I disease in our cohort, and the confounding effect of adjuvant chemotherapy, our data contribute to the growing body of evidence that small T1 HER2+ cancers may have an inferior outcome compared with small HER2-negative cancers (Joerger *et al*, 2011). As evidence continues to emerge that smaller HER2+ cancers may behave more aggressively than previously thought our results add further support to the use of Trastuzumab in these patients.

## Figures and Tables

**Table 1:** Histology of HER2+ patients

*Stage*	
I	92
II	148
III	96
Not pathologically staged	2
	
*Histology*	
Ductal carcinoma	303
Lobular carcinoma	18
Mixed	17
	
*Grade*	
1	7
2	104
3	226
No grade obtainable	1
	
*Hormone status*	
Positive	207
Negative	131

Abbreviation: HER2=human epidermal growth factor receptor 2.

**Table 2:** Proportion of eligible patients treated with Trastuzumab by stage

	**Treated with Trastuzumab (%)**	**Not treated with Trastuzumab (%)**
Stage I	47 (51.1)	45 (49.9)
Stage II	112 (75.7)	36 (24.3)
Stage III	78 (81.3)	18 (18.7)

**Table 3:** Characteristics of treated and untreated stage I patients

	**T1N0—had herceptin**	**T1N0—did not have herceptin**
Number of patients	47	45
T1a (1–5 mm)	5	7
T1b (6–10 mm)	2	10
T1c (11–20 mm)	40	28
Mean tumour size	14.1 mm	12.9 mm
		
Grade 1	0	2
Grade 2	11	19
Grade 3	36	19
HR positive	28	29
HR negative	19	17

Abbreviation: HR=hormone receptor.
